# A retrospective cephalometric study on pharyngeal airway space changes after rapid palatal expansion and Herbst appliance with or without skeletal anchorage

**DOI:** 10.1186/s40510-016-0141-1

**Published:** 2016-09-26

**Authors:** Antonio Manni, Marco Pasini, Maria Rita Giuca, Riccardo Morganti, Mauro Cozzani

**Affiliations:** 1 Private Practice, Racale, Italy; 2 Department of Surgical, Medical and Molecular Pathology and of the Critic Area, University of Pisa, Pisa, Italy; 3 Department of Oncology, Transplants and New Technologies, University of Pisa, Pisa, Italy; 4 Private Practice, La Spezia, Italy; 5 Viale Roma 213, Massa, Massa-Carrara, 54100 Italy

**Keywords:** Airway space, Skeletal anchorage, Class II treatment

## Abstract

**Background:**

The aim of this study is to investigate the pharyngeal airway space changes in patients treated with rapid palatal expansion (RPE) and Herbst appliance with or without skeletal anchorage.

**Methods:**

A 40-patient study group treated with the Herbst RME combination was included; moreover, a comparison between two subgroups based on whether miniscrews were used was evaluated. A subgroup 1 included 20 patients who were treated with RPE and an acrylic splint Herbst with miniscrews, and subgroup 2 included 20 patients who were treated with RPE and an acrylic splint Herbst. A cephalometric analysis was performed before (T1) and after (T2) treatment. The skeletal parameters of the sagittal occlusion analysis of Pancherz were utilized together with some extra measurements to evaluate the airways.

**Results:**

An increased nasopharyngeal airway space was observed in group 1 (*p* < 0.05) from T1 to T2. Furthermore, the increase in nasopharyngeal airway space was significantly higher in subgroup 1 (*p* < 0.05) in comparison to the subgroup 2. Oropharyngeal (OA) and laryngopharyngeal (LA) dimensions were significantly increased in the subgroup 1 at the end of the treatment. In the subgroup 1, a significant decrease in SNA, a significant increase in SNB, and a significant decrease in ANB were observed from T1 to T2. In the subgroup 2, the treatment resulted in a significant decrease in ANB. In both groups, Pogonion increased significantly from T1 to T2.

**Conclusions:**

The results suggest that the RPE and the Herbst appliance allow a slight improvement of the sagittal dimensions of the airways. The oropharyngeal dimension increased significantly more in the skeletal anchorage group.

## Background

The pharyngeal airway space plays a particularly important role in breathing and may regulate the establishment of mouth breathing or nasal breathing [1]. Moreover, it has been suggested that the airway space affects dento-skeletal relationships and facial patterns of growing patients [2]. It was previously reported that retrognathia is associated with airway reduction [3]. Hong et al. found that patients with skeletal class III show a more advanced position of the mandible and increased dimensions of the oropharyngeal space [4]. Moreover, patients with skeletal class II who were treated with mandibular advancement surgery showed an increase in the rear airspace and a widening of the pharyngeal space [5].

Alterations of the airway dimensions can influence the respiration of the subject during the growth phase [6]. It has been reported that a reduction in the blank space of superior airways represents a risk factor, even for obstructive sleep apnea syndrome [7]. Sahoo et al. found that mandibular advancement could be beneficial in reducing airway collapsibility and in preventing sleep disorders due to oropharyngeal airway deficiencies in skeletal class II malocclusion [8]. As regards the upper airways (nasopharyngeal airway space), it was observed a slight increase of the pharyngeal space after rapid palatal expansion therapy; this minimal change is probably due to growth, as in Linder-Aronsson and Leighton’s longitudinal study in 1983, AD2 increased 2.3 mm between the age of 12 and 15 [9].

Moreover, it has been hypothesized that an orthopedic therapy that uses fixed equipment could be used to determine the advancement of the jaw in growing patients, and could positively influence the oropharyngeal and the laryngopharyngeal airway space [10]. In particular, the Herbst appliance is a device for the correction of class II malocclusions in growing patients, which does not require patient compliance and it affects both teeth (i.e., posterior displacement of the upper arch and anterior displacement of the lower arch) and bones (i.e., reduction in the growth of the upper maxillary bone and mandible advancement) [11]. One of the disadvantages of the Herbst appliance, however, is the loss of anchorage in the mandible due to the telescopic forces, which involves a forward inclination of the lower incisors.

Several mechanisms have been proposed to avoid mandibular anchorage loss, among the others, class III elastics and mandibular splints, but these approaches are usually not effective [12]. Recently, innovative methods that are more effective in controlling mandibular arch anchorage and in maintaining the position of the lower incisors have been experimented [13]. In fact, previous studies have shown that the Herbst appliance can be used together with orthodontic miniscrews, anchored into the mandibular bone, in order to ensure better lower incisor anchorage and to reduce flaring of these teeth. It was showed a causal effect between the control of mandibular incisor proclination and the increase in skeletal effects of the Herbst appliance; in fact, a better mandibular incisor proclination control seems to allow a slightly mesial displacement of the mandible [14, 15] and consequently could increased the pharyngeal dimension.

The evaluation of the relationship between the adenoid tissue and the blank space of the upper airways can be measured by different methods. The most common method is the cephalometric analysis of lateral cephalogram, which is a routine examination for orthodontic treatment and has the advantage that it does not require additional radiographs or exposure to further radiation; however, one of the disadvantages of this method is that it evaluates the midsagittal plane, but not the three-dimensional volume [16]. In a previous study, Kinzinger et al. used tracings of lateral cephalograms to measure the posterior airspace in patients treated with a traditional Herbst appliance and did not find any significant changes at the end of the orthodontic treatment [10].

The purpose of this retrospective study was to evaluate whether the pharyngeal airway space increases in patients treated with RPE and a Herbst appliance with or without skeletal anchorage.

## Methods

Sample size calculation was performed. In order to compare the two means with a power of 70, a size of the test of 5 %, a standard deviation of 2.3, and a difference of 1.70, the sample size required 18 patients in each group.

This retrospective study included 40 patients (21 females and 19 males; mean age 12.3 ± 1.5 years) treated with RPE and a Herbst appliance, who met the following inclusion criteria: patients who had lateral cephalogram before and after orthodontic treatment, presence of a permanent dentition or late mixed dentition, presence of a bilateral angle class II division 1 malocclusion, and presence of mandibular deficiency and normal maxilla. Exclusion criteria were presence of serious dental or skeletal malformations, patients with a systemic disease, patients undergoing a drug therapy that may cause skeletal abnormalities, patients with agenesis, patients with premature loss of permanent teeth, the presence of malocclusions in the vertical plane, and patients with poor compliance at check-ups. All procedures were conducted according to the principles expressed in the Declaration of Helsinki.

The patients were divided into two subgroups: subgroup 1 included 20 patients (11 females, 9 males, mean age 12.5 ± 1.7 years), who were consecutively treated with an acrylic splint Herbst appliance with miniscrews, and 20 selected patients (10 females, 10 males, mean age 12.1 ± 1.3 years), who were similar in age and gender to the subgroup 1 and were treated with an acrylic splint Herbst appliance without miniscrews (subgroup 2).

Moreover, the two groups showed a similar degree of malocclusion and skeletal maturation (measured with the cervical vertebral maturation using the classification system of Baccetti et al.) at baseline [17].

Before the Herbst treatment, a rapid palatal expansion was performed in each patient.

In the group of 40 patients, the average expansion time was 15.5 days, and the mean maxillary expansion achieved was 3.1 mm, while the retention period was 6 months.

The average expansion time was 17 days in the subgroup 1, and the mean maxillary expansion achieved was 3.4 mm, while the retention period was 6 months.

In the subgroup 2, the average expansion time was 14 days and the mean maxillary expansion achieved was 2.8 mm, while the retention period was 6 months.

The same orthodontist (AM) treated all patients.

**Fig. 1:**
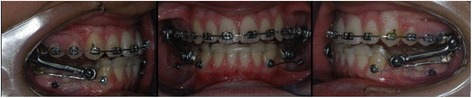
Herbst appliance with skeletal anchorage

For all patients, a total acrylic splint extending from the first lower molar to the first contralateral molar was used to reinforce the anchorage. Further, in the subgroup 1, two miniscrews were included for each patient at the mucogingival junction or at the attached gingiva, between the first mandibular molar and the second premolar. The miniscrews (MAS, Micerium, Avegno, Italy) were 11-mm long, titanium, and shaped like a truncated cone with a diameter of 1.5 or 1.3 mm at the point (according to the bone level) and 2.2 mm at the neck. The shank of the miniscrews was 1 mm in diameter, the threaded part had a length of 8 mm, and the heads featured a hexagonal slot to house the head of the screwdriver or contra-angle hand piece. A metallic ligature, which extended up to the bonded button in the canine of the same hemiarch, was connected to the miniscrew on each side, in order to reinforce mandibular arch anchorage as determined by the telescopic system of Herbst (Fig. 1). The mean duration of orthodontic treatment with the Herbst appliance was 7.95 ± 1.45 months. The mean duration of orthodontic treatment with the Herbst appliance was 7.9 ± 1.4 months in the subgroup 1, and 8 ± 1.5 months in the subgroup 2. During treatment, four miniscrews were replaced, due to excessive mobility, with other four miniscrews that were inserted between the first and second premolar. For each patient, a cephalometric analysis using lateral cephalograms was performed before (T1) and after (T2) orthodontic treatment by the same orthodontist (MP) who was unaware from which group the patient came from. Cephalometric variables are shown in Table 1. In order to fix the hyoid in a consistent position, each patient was requested to breathe in slowly and then exhale, holding the latter position while the film was exposed [18].

**Table 1:** Cephalometric parameters and their descriptions

Maxillary angle SNA	Angle formed by the lines SN and NA (degrees)
Mandibular angle SNB	Angle formed by the lines SN and NB (degrees)
Skeletal class ANB	Angle formed by the lines AN and NB (degrees)
Skeletal divergence SN/GoMe	Angle formed by the lines SN and GoMe (degrees)
Maxillary bone base A/Olp	Distance from the Olp line to point A (mm)
Mandibular bone base Pg/Olp	Distance from the Olp line to the point Pg (mm)
Airway space AD1	Linear distance between the posterior nasal spine and the closest point of adenoidal tissue along the line Ba-PNS (mm)
Airway space AD2	Linear distance between the PNS and the nearest point of adenoid tissue along the line passing through PNS and perpendicular to the line joining S and Ba (mm)
Airway space AD-PtV	Linear distance between the closest point of adenoid tissue and a point on the pterygoid vertical 5 mm above the intersection point between PTV and Ba-PNS (mm)
Oropharyngeal airway space OA	Linear distance of the oropharyngeal space along the palatal plane (PL; plane passing through anterior and posterior nasal spine) passing through the gonion (mm)
Laryngopharyngeal airway space LA	Linear distance of the laryngopharyngeal space along the C4 plane (mm)

**Fig. 2:**
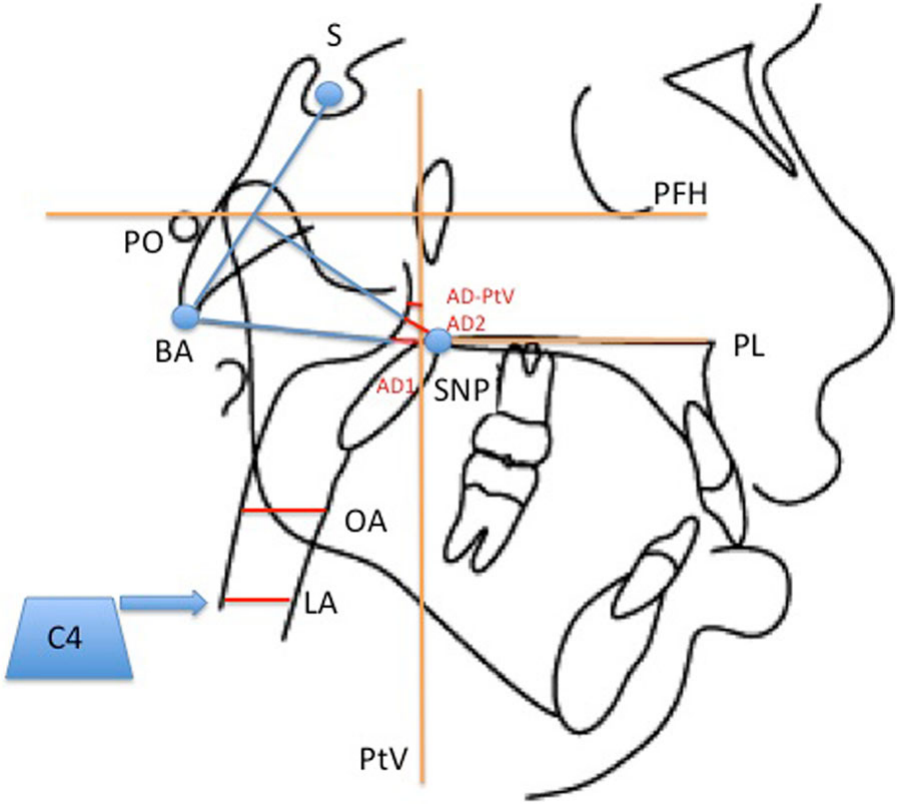
Airways cephalometric analysis. Reference points and lines: sella (*S)*, basion (*BA*), porion (*PO*), posterior nasal spine (*PNS*), Frankfurt plane (*PFH*), pterygoid vertical line (*PtV*), palatal plane (*PL*), the fourth cervical vertebra (*C4*), *AD1*, *AD2*, and *AD-PtV* (upper airway space measurements), oropharyngeal airway space (*OA*), laryngopharyngeal airway space (*LA*)

The parameters analyzed using the cephalometric approach included some parameters used by Woodside, Linder-Aronson, and Lundstrom for evaluating the upper airway of the nasopharynx (Fig. 2) [16, 19].

Oropharyngeal airway space (OA) was measured along the palatal plane (PL), which is a plane parallel to the hard palate passing through the anterior nasal spine (ANS) and the posterior nasal spine (PNS), passing through the anatomical gonion.

Laryngopharyngeal airway space (LA) was measured along the C4 plane (a plane parallel to the PL plane passing through the most inferior anterior point of the fourth cervical vertebra).

**Fig. 3:**
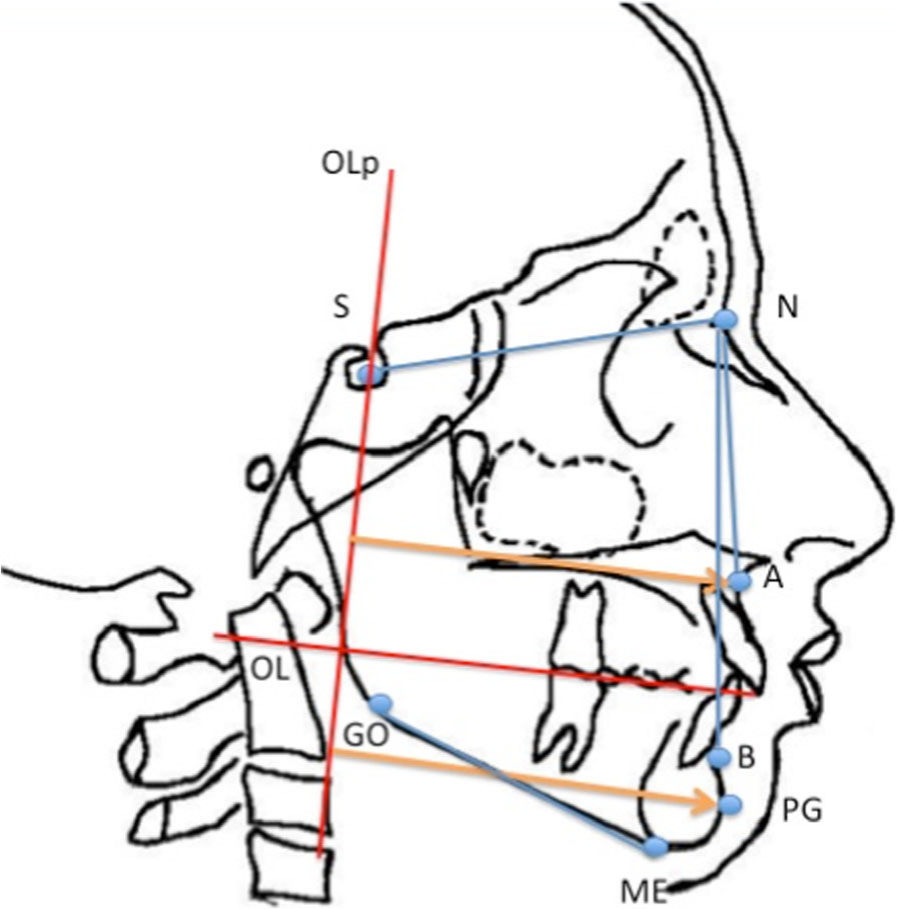
Modified SO-Pancherz analysis. Reference points and lines: sella (*S*), nasion (*N*), subnasal (*A*), supramental (*B*), pogonion (*PG*), gonion (*GO*), menton (*ME*), occlusal line (*OL*), and occlusal line perpendicular (*OLp*)

Moreover, a modified version of the sagittal occlusion (SO) analysis of Pancherz was performed to evaluate, in the sagittal direction, only the position of the jaw [11]. This analysis was carried out by transferring the lines occlusal line (OL) and occlusal perpendicular line (Olp) through the sella from the T1 lateral cephalogram to the T2 lateral cephalogram by superimposing the skeletal stable structures of the anterior cranial base. Then, other cephalometric parameters were considered for the evaluation of the skeletal class (SNA, SNB, ANB) and the skeletal divergence (SN-GoMe) (Fig. 3). All linear and angular measurements were taken to the nearest 0.5 mm and 0.5°, respectively. Dahlberg’s formula [20] was used after measuring each lateral cephalogram twice, with 14 days between each measurement; the method error was less than 0.5 mm and 1°.

### Statistical analysis

Before testing inferential statistics, exploratory phase analyses were performed. The 11 quantitative variables (SNA, SNB, ANB, SN-GoMe, A/Olp, Pg/Olp, AD1, AD2, AD-PtV, OA, and LA) were analyzed using the Kolmogorov-Smirnov test to evaluate whether the data showed a normal distribution. Subsequently, the intra-group and between-group differences were assessed, using both paired and unpaired *t* tests. *p* values <0.05 were considered statistically significant. All statistical analyses were performed using IBM SPSS Statistics V22.0 technology for Windows.

## Results

The results of the intra-group variations from T1 to T2 in the test group of 40 patients are summarized in Table 2.

**Table 2:** Test group (40 patients) before and after treatment

	Test group (T1)	Test group (T2)	*p* value
SNA (degrees)	81.7 ± 4.6	80.8 ± 4.2	0.364
SNB (degrees)	76 ± 3.3	77.1 ± 3.8	0.171
ANB (degrees)	5.6 ± 2.3	3.7 ± 2.2	0.000*
SN-GoMe (degrees)	34.7 ± 5.3	34.2 ± 5.5	0.68
A/Olp (mm)	77 ± 3.6	76.6 ± 3.6	0.621
Pg/Olp (mm)	78.6 ± 6.4	80.3 ± 6.6	0.246
AD1 (mm)	20.6 ± 4	21.6 ± 3.7	0.249
AD2 (mm)	15.2 ± 2.9	16.8 ± 3.1	0.019*
AD-PtV (mm)	8 ± 3	9.3 ± 3.1	0.06
OA (mm)	10.6 ± 2.1	11.9 ± 2.1	0.007*
LA (mm)	11.9 ± 2.5	13.3 ± 2.8	0.02*

*p < 0.05

As regards the two subgroups, the results of the intra-group cephalometric variations from T1 to T2 are summarized in Table 3, and the results of the intergroup cephalometric variations are shown in Table 4.

**Table 3:** Values at T1 (begin of the treatment) and T2 (end of the treatment) in the subgroup 1 and subgroup 2. Intra-group differences

	Subgroup 1 (T1)	Subgroup 1 (T2)	*p* value	Subgroup 2 (T1)	Subgroup 2 (T2)	*p* value
SNA (degrees)	81.1 ± 4.6	80.2 ± 4.5	0.025*	82.3 ± 4.7	81.5 ± 3.9	0.163
SNB (degrees)	75.3 ± 3.6	76.6 ± 3.9	0.004*	76.8 ± 3.1	77.6 ± 2.7	0.061
ANB (degrees)	5.8 ± 2.4	3.5 ± 2.2	0.000*	5.5 ± 2.3	3.9 ± 2.3	0.002*
SN-GoMe (degrees)	35 ± 5.9	34 ± 5.7	0.078	34.4 ± 4.7	34.9 ± 5.4	0.364
A/Olp (mm)	78.5 ± 3.6	77.7 ± 3.7	0.119	75.5 ± 3.7	75.6 ± 3.6	0.810
Pg/Olp (mm)	80.3 ± 6.1	82.6 ± 6.8	0.005*	77 ± 6.8	78 ± 6.4	0.027*
AD1 (mm)	21.4 ± 4.2	22.5 ± 3.8	0.160	19.9 ± 3.8	20.8 ± 3.7	0.067
AD2 (mm)	15.1 ± 3.1	17.5 ± 3	0.000*	15.3 ± 2.7	16.2 ± 3.2	0.009*
AD-PtV (mm)	8 ± 3.5	9.7 ± 3.7	0.000*	8.1 ± 2.5	9 ± 2.5	0.013*
OA (mm)	11.2 ± 2.3	12.9 ± 2.5	0.000*	10.1 ± 1.9	11 ± 1.8	0.168
LA (mm)	11.7 ± 2.2	13.4 ± 2.9	0.000*	12.2 ± 2.8	13.2 ± 2.8	0.061

*p < 0.05

**Table 4:** Differences between T2 (end of therapy) and T1 (begin of therapy) in the two subgroups. Between-groups differences

	Subgroup 1 T2–T1	Subgroup 2 T2–T1	*p* value
SNA (degrees)	−0.9 ± 1.6	−0.8 ± 2.5	0.88
SNB (degrees)	1.3 ± 1.7	0.8 ± 1.7	0.36
ANB (degrees)	−2.3 ± 1.1	−1.6 ± 2	0.18
SN-GoMe (degrees)	−1 ± 2.5	0.5 ± 2.2	0.05
A/Olp (mm)	−0.8 ± 2.2	0.1 ± 1.8	0.17
Pg/Olp (mm)	2.3 ± 3.3	1 ± 1.9	0.13
AD1 (mm)	1.1 ± 3.5	0.9 ± 1.9	0.74
AD2 (mm)	2.4 ± 1	0.9 ± 1.4	0.00*
AD-PtV (mm)	1.7 ± 1.4	0.9 ± 1.4	0.07
OA (mm)	1.7 ± 1.2	0.9 ± 0.6	0.01*
LA (mm)	1.65 ± 3.6	1 ± 2.2	0.49

*p < 0.05

### Airway analysis

In the test group of 40 patients, a significant (*p* < 0.05) increase of AD2 was observed after the treatment. Moreover, a significant (*p* < 0.05) increase of OA and LA was found at T2. An increase of AD1 and AD/PtV was observed at T2; however, the difference was not significant (*p* > 0.05).

An increased nasopharyngeal airway space was observed in subgroup 1: in particular, AD2 (linear distance between the PNS and the nearest point of adenoid tissue along the line passing through PNS and perpendicular to the line joining S and Ba) and AD-PtV (linear distance between the closest point of adenoid tissue and a point on the pterygoid vertical 5 mm above the intersection point between PTV and Ba-PNS) increased significantly (*p* < 0.05) from T1 to T2, and AD1 (linear distance between the posterior nasal spine and the closest point of adenoidal tissue along the line Ba-PNS) also increased, but not significantly (*p* > 0.05). Similarly, in the subgroup 2, an increase in the nasopharyngeal airway space was found after treatment: AD2 and AD-PtV increased significantly (*p* < 0.05), and AD1 also increased, but not significantly (*p* > 0.05). A comparison of subgroup 1 with subgroup 2 showed that the increase in nasopharyngeal airway space is significantly higher in subgroup 1 for AD2 (*p* < 0.05). However, no statistically significant difference was found between the two groups with regard to AD1 or AD-PtV values.

In subgroup 1, a significant increase of OA and LA was observed at the end of Herbst treatment (*p* < 0.05). Moreover, also in subgroup 2, an increase in parameters OA and LA was found at the end of the treatment, but the difference was not significant (*p* > 0.05).

A comparison of the two groups showed no statistically difference with regard to LA (*p* > 0.05), while OA was statistically higher in the subgroup 1 in comparison to the subgroup 2 (*p* < 0.05).

### Skeletal class, position of the maxillary, and skeletal divergence

In the test group of 40 patients, a significant decrease (*p* < 0.05) of ANB was observed at the end of the treatment. A slight decrease of SNA, an increase of SNB, and a slight decrease of SN-GoMe were found, but the difference was not significant (*p* > 0.05). A non-significant (*p* > 0.05) decrease of A/Olp and increase of Pg/Olp were observed after the treatment.

In subgroup 1 treated with the Herbst appliance with skeletal anchorage, a significant decrease (*p* < 0.05) in parameter SNA, a significant increase (*p* < 0.05) in parameter SNB, and a significant decrease (*p* < 0.05) in parameter ANB were observed from T1 to T2. In subgroup 2 treated with the Herbst appliance without skeletal anchorage, the treatment resulted in a decrease of SNA that was not significant (*p* > 0.05), an increase of SNB that was not significant (*p* > 0.05), and a significant decrease (*p* < 0.05) of ANB from T1 to T2. In both groups, the sagittal position of point A showed a retraction at the end of orthodontic treatment, which was not statistically significant in either group (*p* > 0.05). In contrast, the distance from the Olp line to Pg increased significantly (*p* < 0.05) after treatment in both groups. In subgroup 1, SN-GoMe showed a slight decrease, which was not significant (*p* > 0.05), from T1 to T2 with a counterclockwise rotation of the mandible. In subgroup 2, a slight, non-significant (*p* > 0.05), increase in skeletal divergence with a mandibular clockwise rotation was observed at T2. When comparing the two groups, no statistically significant differences were found (*p* > 0.05) for any of the parameters, and the mandibular divergence parameter was found to be of borderline significance.

## Discussion

Orthodontic therapy with RPE and Herbst resulted in a slight increase of pharyngeal dimension. In particular, the increase of the nasopharyngeal space was probably related to growth, and the increase of the oro- and laryngopharyngeal dimensions was probably related to the forward shift of the mandible.

In fact, orthodontic treatment with the Herbst appliance with or without skeletal anchorage resulted in effective improvements in class II patients, as determined by the retraction and/or arrest of upper jaw growth, and the development of mandibular growth [12]. The orthopedic changes were slightly more significant in subgroup 1 than in subgroup 2, which is probably related to the fact that a stronger dental anchorage can allow for greater forward displacement of the jaw, as suggested in the previous studies [14, 15]. Furthermore, it appears that, in addition to mandibular growth, there is a slight increase in anterior mandibular rotation in subgroup 1 treated with the Herbst appliance and skeletal anchorage.

The oropharyngeal and laryngopharyngeal airways, in the subgroup 1, showed an increase in size in the sagittal direction that could be related to orthopedic changes caused by the Herbst appliance and in part to the forward shift of the jaw. The subgroup 1 showed a higher increase of OA, at the end of the therapy, which is probably related to the skeletal anchorage, which maintains the lower incisor anteroposterior position and facilitates a forward displacement of the lower jaw.

Indeed, it has been observed that the air volume is directly related to the position of the jaw, as discussed by Kikuchi [21]. Therefore, orthodontic treatment of class II with correction of the position of the mandible could be particularly beneficial for those patients with respiratory problems, such as prevention of obstructive sleep apnea syndrome [22].

By analyzing the airway, it was found that there is a slight greater increase in parameter AD2 and for the sum of upper airways parameters in subgroup 1. This could be related to the slight greater palatal expansion that was performed in subgroup 1 in comparison to subgroup 2. Moreover, the increase of nasopharyngeal dimension after rapid palatal expansion, observed in the present study, is similar to the findings of [9].

The forward displacement of the jaw, particularly after surgical procedures, has been highlighted in the literature for improving the upper airways [5]. However, few studies have analyzed the effects of the Herbst appliance on airways, and, to our knowledge, no study to date has analyzed the airways of patients treated with the Herbst appliance with skeletal anchorage. Battagel et al. analyzed the pharyngeal space of patients with obstructive sleep apnea syndrome at rest and in maximum comfortable protrusion, and found some airway improvements related to mandibular protrusion [7].

Iwasaki et al, using measurements from three-dimensional cone-beam computed tomography images of the pharyngeal airway, observed that Herbst appliance increases both the oropharyngeal and the laryngopharyngeal dimensions with similar results [23]. Furthermore, also in a recent study of Koay et al. it was found, from lateral cephalograms, that the Herbst appliance increased the oropharyngeal and hypopharyngeal airway dimensions among patients with class II malocclusion [24].

An ideal study design to evaluate airway dimension after orthodontic treatment would also include a control group of untreated patients in order to observe purely growth changes. However, lateral cephalogram of patients without any orthodontic treatment were not available for this study and could not be taken for ethical reasons.

Mislik et al. measured the pharyngeal space and the physiological modifications based on a large sample size of lateral cephalograms of untreated patients and stated that the airway measurements are being established in early childhood; only a slight continuous increase of about 1 mm was detected between 6 and 17 years of age [25].

So, we can hypothesize that the changes in airway dimension related purely to growth, in our study, were limited due to the short treatment time.

Furthermore, a limitation of the current study, in addition to the small number of patients, is the cephalometric analysis of lateral cephalogram method, which only allows a two-dimensional evaluation of the airway and cannot be used to determine the thickness and volume of the pharyngeal airway space [26, 27]. Currently, the cone-bean computed tomography (CBCT) method provides an accurate three-dimensional analysis of the airways, and on the other hand, CBCT cannot be performed in all patients for legal and ethical reasons, while lateral cephalogram is a legal and ethical accepted method utilized routinely in all orthodontic patients. Moreover, Kaur et al. have observed that the cephalometric analysis of the lateral cephalogram is a reliable and reproducible method for evaluating the pharyngeal space [2].

In the present study, lateral cephalogram was taken fixing the hyoid in a consistent position in order to obtain a reliable analysis for each patient [18].

Therefore, further studies evaluating a larger number of patients and, possibly, the three-dimensional evaluation of the airways using CBCT, where legally and ethically acceptable, could be useful to assess more precisely the possible correlations between orthodontic treatments and airway space.

## Conclusions

The results of this retrospective study showed that the Herbst appliance is able to correct the malocclusion of skeletal class II and allows a slight improvement of the sagittal dimensions of the oro- and laryngopharyngeal airways. When equipped with orthodontic miniscrews that reinforce mandibular anchorage, the Herbst appliance enhanced orthopedic effects and allowed a slight increase of oropharyngeal airway space.

## References

[1] Fastuca R, Zecca PA, Caprioglio A (2014). Role of mandibular displacement and airway size in improving breathing after rapid maxillary expansion. Prog Orthod.

[2] Kaur S, Rai S, Kaur M (2014). Comparison of reliability of lateral cephalogram and computed tomography for assessment of airway space. Niger J Clin Pract.

[3] Cossellu G, Biagi R, Sarcina M, Mortellaro C, Farronato G (2015). Three-dimensional evaluation of upper airway in patients with obstructive sleep apnea syndrome during oral appliance therapy. J Craniofac Surg.

[4] Hong JS, Oh KM, Kim BR, Kim YJ, Park YH (2011). Three-dimensional analysis of pharyngeal airway volume in adults with anterior position of the mandible. Am J Orthod Dentofacial Orthop.

[5] Kochel J, Meyer-Marcotty P, Sickel F, Lindorf H, Stellzig-Eisenhauer A (2013). Short-term pharyngeal airway changes after mandibular advancement surgery in adult class II-Patients—a three-dimensional retrospective study. J Orofac Orthop.

[6] Jeans WD, Fernando DC, Maw AR, Leighton BC (1981). A longitudinal study of the growth of the nasopharynx and its contents in normal children. Br J Radiol.

[7] Battagel JM, Johal A, L'Estrange PR, Croft CB, Kotecha B (1999). Changes in airway and hyoid position in response to mandibular protrusion in subjects with obstructive sleep apnoea (OSA). Eur J Orthod.

[8] Sahoo NK, Jayan B, Ramakrishna N, Chopra SS, Kochar G (2012). Evaluation of upper airway dimensional changes and hyoid position following mandibular advancement in patients with skeletal class II malocclusion. J Craniofac Surg.

[9] Linder-Aronson S, Leighton BC (1983). A longitudinal study of the development of the posterior nasopharyngeal wall between 3 and 16 years of age. Eur J Orthod.

[10] Kinzinger G, Czapka K, Ludwig B, Glasl B, Gross U, Lisson J (2011). Effects of fixed appliances in correcting Angle Class II on the depth of the posterior airway space: FMA vs. Herbst appliance—a retrospective cephalometric study. J Orofac Orthop.

[11] Pancherz H (1982). The mechanism of class II correction in Herbst appliance treatment. A cephalometric investigation. Am J Orthod.

[12] Weschler D, Pancherz H (2005). Efficiency of three mandibular anchorage forms in Herbst treatment: a cephalometric investigation. Angle Orthod.

[13] Jv B, Ludwig B, Ruf S (2015). Anchorage loss due to Herbst mechanics-preventable through miniscrews?. Eur J Orthod.

[14] Manni A, Mutinelli S, Pasini M, Mazzotta L, Cozzani M (2016). Herbst appliance anchored to miniscrews with 2 types of ligation: effectiveness in skeletal Class II treatment. Am J Orthod Dentofacial Orthop.

[15] Manni A, Pasini M, Mazzotta L, Mutinelli S, Nuzzo C, Grassi FR (2014). Comparison between an acrylic splint Herbst and an acrylic splint miniscrew-Herbst for mandibular incisors proclination control. Int J Dent.

[16] Galvao MAB, Almeida MAO (2010). Comparison of two extraoral radiographic techniques used for nasopharyngeal airway space evaluation. Dental Press J Orthod.

[17] Baccetti T, Franchi L, James A, McNamara J (2005). The cervical vertebral maturation (CVM) method for the assessment of optimal treatment timing in dentofacial orthopedics. Semin Orthod.

[18] Battagel JM, Johal A, Kotecha B (2000). A cephalometric comparison of subjects with snoring and obstructive sleep apnoea. Eur J Orthod.

[19] Linder-Aronson S, Henrikson CO (1973). Radiocephalometric analysis of anteroposterior nasopharyngeal dimensions in 6- to 12-year-old mouth breathers compared with nose breathers. ORL J Otorhinolaryngol Relat Spec.

[20] Dahlberg G (1940). Statistical methods for medical and biological students.

[21] Kikuchi Y (2008). Three-dimensional relationship between pharyngeal airway and maxillo-facial morphology. Bull Tokyo Dent Coll.

[22] Ghodke S, Utreja AK, Singh SP, Jena AK (2014). Effects of twin-block appliance on the anatomy of pharyngeal airway passage (PAP) in class II malocclusion subjects. Prog Orthod.

[23] Iwasaki T, Takemoto Y, Inada E, Sato H, Saitoh I, Kakuno E (2014). Three-dimensional cone-beam computed tomography analysis of enlargement of the pharyngeal airway by the Herbst appliance. Am J Orthod Dentofacial Orthop.

[24] Koay WL, Yang Y, Tse CS, Gu M (2016). Effects of two-phase treatment with the Herbst and preadjusted edgewise appliances on the upper airway dimensions. Scientific World Journal.

[25] Mislik B, Hänggi MP, Signorelli L, Peltomäki TA, Patcas R (2014). Pharyngeal airway dimensions: a cephalometric, growth-study-based analysis of physiological variations in children aged 6-17. Eur J Orthod.

[26] Ucar FI, Uysal T (2012). Comparision of orofacial airway dimensions in subject with different breathing pattern. Prog Orthod.

[27] Edwards R, Alsufyani N, Heo G, Flores-Mir C (2014). The frequency and nature of incidental findings in large-field cone beam computed tomography scans of an orthodontic sample. Prog Orthod.

